# SNP-based real-time pyrosequencing as a sensitive and specific tool for identification and differentiation of *Rickettsia* species in *Ixodes ricinus* ticks

**DOI:** 10.1186/1471-2334-12-261

**Published:** 2012-10-18

**Authors:** Elisabeth Janecek, Sabine Streichan, Christina Strube

**Affiliations:** 1 Institute for Parasitology, University of Veterinary Medicine Hannover, Buenteweg 17, Hannover 30559, Germany

**Keywords:** *Rickettsia helvetica*, *Rickettsia monacensis*, *Rickettsia massiliae*, *Rickettsia felis*, *Ixodes ricinus*, Diagnostic, Sequencing

## Abstract

**Background:**

Rickettsioses are caused by pathogenic species of the genus *Rickettsia* and play an important role as emerging diseases. The bacteria are transmitted to mammal hosts including humans by arthropod vectors. Since detection, especially in tick vectors, is usually based on PCR with genus-specific primers to include different occurring *Rickettsia* species, subsequent species identification is mainly achieved by Sanger sequencing. In the present study a real-time pyrosequencing approach was established with the objective to differentiate between species occurring in German *Ixodes* ticks, which are *R. helvetica*, *R. monacensis*, *R. massiliae*, and *R. felis*. Tick material from a quantitative real-time PCR (qPCR) based study on *Rickettsia*-infections in *I. ricinus* allowed direct comparison of both sequencing techniques, Sanger and real-time pyrosequencing.

**Methods:**

A sequence stretch of rickettsial citrate synthase (*gltA*) gene was identified to contain divergent single nucleotide polymorphism (SNP) sites suitable for *Rickettsia* species differentiation. Positive control plasmids inserting the respective target sequence of each *Rickettsia* species of interest were constructed for initial establishment of the real-time pyrosequencing approach using Qiagen’s PSQ 96MA Pyrosequencing System operating in a 96-well format. The approach included an initial amplification reaction followed by the actual pyrosequencing, which is traceable by pyrograms in real-time. Afterwards, real-time pyrosequencing was applied to 263 *Ixodes* tick samples already detected *Rickettsia*-positive in previous qPCR experiments.

**Results:**

Establishment of real-time pyrosequencing using positive control plasmids resulted in accurate detection of all SNPs in all included *Rickettsia* species. The method was then applied to 263 *Rickettsia*-positive *Ixodes ricinus* samples, of which 153 (58.2%) could be identified for their species (151 *R. helvetica* and 2 *R. monacensis*) by previous custom Sanger sequencing. Real-time pyrosequencing identified all Sanger-determined ticks as well as 35 previously undifferentiated ticks resulting in a total number of 188 (71.5%) identified samples. Pyrosequencing sensitivity was found to be strongly dependent on *gltA* copy numbers in the reaction setup. Whereas less than 10^1^ copies in the initial amplification reaction resulted in identification of 15.1% of the samples only, the percentage increased to 54.2% at 10^1^-10^2^ copies, to 95.6% at >10^2^-10^3^ copies and reached 100% samples identified for their *Rickettsia* species if more than 10^3^ copies were present in the template.

**Conclusions:**

The established real-time pyrosequencing approach represents a reliable method for detection and differentiation of *Rickettsia* spp. present in *I. ricinus* diagnostic material and prevalence studies. Furthermore, the method proved to be faster, more cost-effective as well as more sensitive than custom Sanger sequencing with simultaneous high specificity.

## Background

*Rickettsia* spp. are Gram-negative, obligate intracellular bacteria with pleomorphic appearance [1]. The life cycle involves arthropod vectors like ticks, fleas, lice and mites, which are of great importance for the natural retention of the pathogen [2]. Tick-borne rickettsioses are originating from species belonging to the spotted fever group [3]. The most abundant European tick species *Ixodes ricinus* serves as vector for the potentially pathogenic *Rickettsia* species *R. helvetica* and *R. monacensis*[4-7]. Additionally, *R. massiliae* as well as *R. felis* has been detected in *I. ricinus* in one case each [5]. However, no vector competences are proven for those species.

*R. helvetica* is connected to “summer flu”, a feverish infection without rash [5,8], but also meningitis and perimyocarditis [9,10]. By contrast, *R. monacensis, R. massiliae*, and *R. felis* cause the classical form of spotted fever [5]. Even though Boretti et al. [11] describe a species-specific real-time PCR for *R. helvetica* based on the 23S rRNA gene, *Rickettsia* detection in *Ixodes* ticks is mainly based on conventional or quantitative real-time PCR (qPCR) with genus- but not species-specific primers to include the different *Rickettsia* species followed by species differentiation via DNA sequencing with the classical chain termination by Sanger.

A more recent sequencing technology is pyrosequencing, which is based on the principle that nucleotide incorporation in a DNA strand is accompanied by release of pyrophosphate. This is converted into ATP by ATP sulfurylase in the presence of adenosine 5' phosphosulfate. ATP, in turn, drives conversion of luciferin to oxyluciferin that emits visible light. This light signal is visualized as a peak, whose height is proportional to the number of nucleotides incorporated. To prevent incorrect detection, unincorporated nucleotides and ATP are degraded by apyrase before another nucleotide is added.

In the present study, a single nucleotide polymorphism (SNP)-based real-time pyrosequencing approach was evaluated as an in-lab diagnostic tool for detection and differentiation of *Rickettsia* spp. in *I. ricinus*. The used PSQ 96MA Pyrosequencing System (Qiagen, formerly Biotage) displays the peaks of incorporated nucleotides in real time in the so called pyrogram.

## Results

### Establishment of real-time pyrosequencing

**Figure 1:**
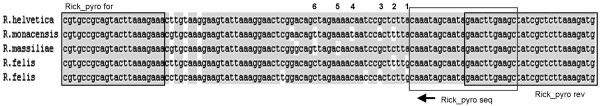
**SNPs in the rickettsial *gltA *gene allowing species differentiation by real-time pyrosequencing.** Used primers are marked by boxes. Note that Rick_pyro rev and Rick_pyro seq consist of complementary nucleotides and thus the lagging strand is sequenced. Detected SNPs are indicated by numbers. The displayed alignment is on GenBank accession numbers DQ910785 (*R. helvetica*), GQ925820 (*R. monacensis*), DQ459393 (*R. massiliae*), U33922 and GQ255903 (*R. felis*).

**Figure 2:**
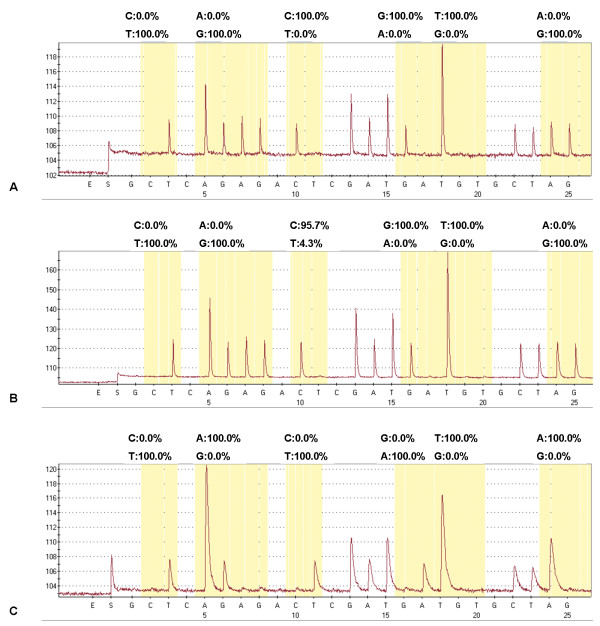
***Rickettsia *real-time pyrograms.** Figure 2a and 2b show the pyrograms of ticks infected with *R. helvetica*, whereas Figure 2c shows the pyrogram of a tick infected with *R. monacensis*.

In real-time generated pyrograms revealed that in all SNP positions (Figure 1) of all *Rickettsia* positive control plasmids (*R. helvetica, R. massiliae*, and *R. monacensis*, and two *R. felis* plasmids) the correct nucleotide was incorporated exclusively (exemplarily see Figure 2a and 2c), thus providing specific *Rickettsia* spp. differentiation. As evident from Figure 1, the first SNP position already delimits *R. felis* (nucleotide “T”) from the remaining species, which contain the nucleotide “C” at this position. The fourth SNP is specific to *R. monacensis* and SNP 5 to *R. massiliae*, whereas *R. helvetica*-specificity is based on the combination of SNP 1 and 6.

### Pyrosequencing of *Rickettsia*-positive tick DNA

Whilst Sanger sequencing identified 151 (57.4%) and 2 (0.8%) out of the 263 available ticks to be infected with *R. helvetica* and *R. monacensis*, respectively, the established real-time pyrosequencing confirmed all Sanger-identified tick samples and additionally detected *R. helvetica* in 35 (31.8%) of 110 previously unidentified samples. So the overall real-time pyrosequencing *Rickettsia* differentiation rate was 71.5% (188/263), whereas custom Sanger sequencing identified 58.2% (153/263) tick samples only. This difference was statistically significant (P= 0.002). Detailed information on statistical results is given in Table 1.

**Table 1:** Comparison of Sanger sequencing and real-time pyrosequencing

**Sensitivity**			
**Copy number in initial template (2 μl)**	**Sanger Sequencing**	**real-time pyrosequencing**	**P-value**
	**Number of identified tick samples (%)**		
<10^1^	1/73 (1.4)	11/73 (15.1)	0.013*
10^1^-10^2^	5/24 (8.3)	13/24 (54.2)	0.180
>10^2^-10^3^	28/45 (62.2)	43/45 (95.6)	0.239
>10^3^-10^4^	72/74 (97.3)	74/74 (100)	0.999
>10^5^-10^5^	42/42 (100)	42/42 (100)	1.000
>10^5^-10^6^	4/4 (100)	4/4 (100)	1.000
>10^6^	1/1 (100)	1/1 (100)	1.000
all samples	153/263 (58.2)	188/263 (71.5)	0.002*

* Statistically significant.

Sensitivity of pyrosequencing increased with *gltA* copy numbers: if >10^2^-10^3^ copies were present in 2 μl template of the initial amplification reaction, the detection rate was 95.6% (43/45 tick samples), and reached 100% (121/121) in presence of more than 10^3^*gltA* copies. *Rickettsia* species differentiation was possible only in 24.7% of the low-burden ticks, divided into a differentiation rate of 15.1% (11/73 tick samples) in case of less than 10^1^ copies/template and 54.2% (13/24) in case of 10^1^-10^2^ copies. All but two tick samples identified for their *Rickettsia* species by real-time pyrosequencing but not custom Sanger sequencing contained less than 10^3^*gltA* copies/2 μl template. In 198 of the 263 samples (75.3%), at least one SNP position showed a proportion of less than 100% for a specific nucleotide (exemplarily shown in Figure 2b). Detailed sensitivity comparison of both methods is shown in Table 1.

## Discussion

Rickettsioses play an important role as emerging diseases, so there is a need for appropriate diagnostic tools. Our results show that real-time pyrosequencing is a specific and very sensitive diagnostic tool for differentiation between *Rickettsia* species occurring in *I. ricinus* in Europe.

Specificity of the established *Rickettsia* real-time pyrosequencing was validated by use of positive control plasmids containing the *gltA* target sequence of the individual *Rickettsia* species of interest. Even if detection of “exclusive” or other SNPs was not 100% (cf. Figure 2b), the combination of the remaining SNPs enabled *Rickettsia* species differentiation. Application to diagnostic samples, which were DNA samples of *I. ricinus* ticks naturally infected with rickettsiae, showed superior sensitivity of real-time pyrosequencing compared to custom Sanger sequencing results from a previous study [12].

Not unexpectedly, real-time pyrosequencing sensitivity was strongly dependent on *gltA* copy numbers revealed by qPCR [12]. Low copy numbers (≤10^2^) in the initial amplification reaction often resulted in either weak or missing bands in gel electrophoresis with the consequence of insufficient template for subsequent pyrosequencing. Notably, all but two tick samples identified for their *Rickettsia* species by real-time pyrosequencing but not custom Sanger sequencing contained less than 10^3^*gltA* copies/2 μl template demonstrating that superior sensitivity of real-time pyrosequencing is due to successful sequencing of lower target copy numbers.

Detection of less than 100% for a specific nucleotide may be due to incorrect signaling, since double infections (two *Rickettsia* species in one tick) do not seem to occur [13] and have yet to be observed (upon request the manufacturer’s technical support stated that 5% incorrect signaling is in the range of tolerance). Nevertheless, identification of the *Rickettsia* species (*R. helvetica* in all cases) was possible based on the remaining SNP positions (see above).

## Conclusions

We have developed the first real-time pyrosequencing approach for *Rickettsia* species. The method provides useful in-lab detection and species identification in diagnostic (tick) material as well as prevalence studies. Since rickettsioses gain attention as emerging diseases, there is a need for appropriate diagnostic tools. Real-time pyrosequencing proved to be faster, more cost-effective as well as more sensitive than custom Sanger sequencing. In detail, *Rickettsia* species identification by pyrosequencing usually takes about an hour, which includes preparation of samples as well as the actual sequencing whereas results of custom Sanger sequencing lasts at least until the next day. Additionally, the entire pyrosequencing procedure (initial PCR and pyrosequencing reaction itself) using Qiagen’s PSQ 96MA sequencer does not exceed 1.50 € per sample. Thus, it is an optimal completion of studies detecting the genus *Rickettsia* but not individual species.

## Methods

### Selection of a target sequence stretch containing suitable SNPs

A sequence stretch of the rickettsial citrate synthase (*gltA*) gene was identified to contain divergent SNP sites suitable for species differentiation of *R. helvetica, R. massiliae, R. monacensis*, and *R. felis*. Thereby, SNP selection was based on sequences published in the GenBank database [accession nos. DQ910785, DQ131912, RHU59723, EU359285, EU359286, EU359287 (*R. helvetica*); GQ925820, GQ925822, DQ100163, EU665235, EU665236 (*R. monacensis*); DQ459393, DQ212705, region 1303919–1305247 of CP000683 (*R. massiliae*); as well as U33922, GQ255903, and region 1424392–1425699 of CP000053 (*R. felis*)].

### Positive control plasmids

Positive control plasmids containing the respective target sequence of each included *Rickettsia* species was constructed using overlapping primers, whose sequences are listed in Table 2. PCR set up was as follows: 20.5 μl ddH_2_O, each 0.5 μl forward and reverse primer (10 pmol), 0.5 μl dNTPs (10 mM each), 2.5 μl 10x buffer and 0.5 μl Advantage 2 Polymerase Mix (Clontech). Cycling conditions consisted of 95°C for 1 min, 35 cycles at 95°C for 30 sec, 55°C for 1 min and 72°C for 30 sec followed by final elongation at 72°C for 10 min. Amplification products were inserted into the PCR®4-TOPO® vector (TOPO TA Cloning® Kit for Sequencing, Life Technologies) and transformed into One Shot® TOP10 chemically competent *E. coli* (Life Technologies). Replicated plasmid DNA was extracted with the NucleoSpin® Plasmid kit (Macherey-Nagel) and accuracy of the rickettsial insert sequences was verified by sequencing (Seqlab Sequencing Laboratories).

**Table 2:** **Overlapping primers for *Rickettsia *positive control plasmids**

	**Forward primer**	**Reverse primer**
*R. helvetica*	5’-CGTGCCGCAGTACTTAAAGAAACTTGTAAGGAAGTA TTAAAGGAACTCGGACAGCTAGAAAACAATCCGC-3’	5’-CATCTTTAAGAGCGATAGCTTCAAGTTCTATTGCT ATTTGTAAGAGCGGATTGTTTTCTAGCTGTCCGAG-3’
*R. monacensis*	5’-CGTGCCGCAGTACTTAAAGAAACGTGCAAAGAAGT ATTAAAGGAACTCGAACAGTTAGAAAATAATCCAC-3’	5’-CATCTTTAAGAGCGATAGCTTCAAGTTCTATTGCT ATTTGTAAAAGTGGATTATTTTCTAACTGTTCGAG-3’
*R. massiliae*	5’-CGTGCCGCAGTACTTAAAGAAACGTGCAAAGAAGT ATTAAAGGAACTCGGGCAGTTAGACAACAATCCGC-3’	5’-CATCTTTAAGAGCGATAGCTTCAAGTTCTATTGCT ATTTGTAAGAGCGGATTGTTGTCTAACTGCCCGAG-3’
*R. felis* 1	5’-CGTGCCGCAGTACTTAAAGAAACCTGCAAAGAAGT ATTAAAGGAACTTGGACAGCTAGAAAACAACCCAC-3’	5’-CATCTTTAAGAGCGATAGCTTCAAGTTCTATTGCT ATTTGCAAGAGTGGGTTGTTTTCTAGCTGTCCAAG-3’
*R. felis* 2	5’-CGTGCCGCAGTACTTAAAGAAACTTGCAAAGAAGT ATTAAAGGAACTCGGACAGCTAGAAAACAATCCGC-3’	5’-CATCTTTAAGAGCGATAGCTTCAAGTTCTATTGCT ATTTGCAAAAGCGGATTGTTTTCTAGCTGTCCGAG-3’

### Primer design for pyrosequencing

Primers for real-time-pyrosequencing were designed using the Pyrosequencing™ Assay Design software (version 1.0; Biotage). Primer positions within the target sequence and detected SNPs of the different *Rickettsia* species are shown in Figure 1. It should be noted that the pyrosequencing primer Rick_pyro seq sequences the lagging strand.

### Establishment of real-time pyrosequencing

For initial establishment, each 2 μl *Rickettsia* positive control plasmid DNA served as template in an initial amplification reaction consisting of 39.5 μl ddH_2_O, 5 μl 10x buffer, 0.5 μl PerfectTaq Polymerase (5’PRIME), 1 μl 5’-biotinylated Rick_pyro for, 1 μl Rick_pyro rev (10 pmol each; Sigma-Aldrich), and 1 μl dNTPs (10 mM each). Thermocycling was as follows: 95°C for 3 min followed by 45 cycles of 95°C, 60°C and 72°C for each 30 seconds and final elongation at 72°C for 10 minutes. Products were checked by gel electrophoresis. Distinct bands at 115 bp allowed further processing with the PSQ 96MA Pyrosequencing System (Qiagen), which operates in a 96 well format. First, 40 μl biotinylated PCR product was immobilized to 4 μl Streptavidin Sepharose™ beads (GE Healthcare) with 40 μl binding buffer (10 mM HCl, 1 mM EDTA, 2 M NaCl, 0.1% Tween 20, pH 7.6) for 10 minutes with vigorous shaking. The resulting complex was captured with the PyroMark Vacuum Prep Tool (Qiagen) and washed with 70% ethanol for 5 seconds. Strand separation was achieved by treatment with denaturing buffer (0.2 M NaOH) for 5 seconds followed by 10 seconds washing with 10 mM Tris-acetate buffer, pH 7.6. Afterwards, the biotinylated single strands were transferred into a PSQ 96 Plate Low containing 40 μl annealing buffer (20 mM Tris-acetate, 2 mM MgAC_2_) and 0.4 μM sequencing primer (Rick_pyro seq). Following incubation for 2 minutes at 80°C using the PSQ 96 Sample Prep Thermoplate Low, required real-time pyrosequencing reagents (nucleotides as well as enzyme- and substrate mixtures; Pyro Mark® Gold Q96 Reagents, Qiagen) were added to the PSQ 96 Reagent Cartridge according to the volume information given by the instrument’s software. The sequence to analyze entered into the PyroMark ID software was: C/T AA A/G AG C/T GGATT G/A TT T/G TCTA G/A C. The nucleotide dispensation order to differentiate between the *Rickettsia* spp. included blank controls and was as follows: GCTCAGAGACTCGATGATGTGCATG (cf. Figure 2). Incorporated nucleotides were visualized in real-time pyrograms.

### Real-time pyrosequencing of *Rickettsia*-infected ticks

To apply the established real-time pyrosequencing to diagnostic samples, *I. ricinus* tick DNA samples were evaluated. In a previous qPCR study, 363 questing *I. ricinus* DNA samples were found positive for *Rickettsia* spp. by qPCR [12], of which a subset of 263 samples was available for the present study. The samples comprised all developmental tick stages (larvae, nymphs as well as female and male adults) and included different numbers of *gltA* copies per 2 μl templates: 73 tick samples contained less than 10^1^ copies; 24 samples contained 10^1^-10^2^ copies; 45 samples >10^2^-10^3^ copies; 74 samples >10^3^-10^4^ copies; 42 samples >10^5^-10^5^ copies; 4 samples >10^5^-10^6^ copies; and one sample contained >10^6^ copies. Tick samples were amplified and processed as described above. Amplification products showing no or weak bands only in gel electrophoresis were re-amplified before further processing with actual real-time pyrosequencing. In each run, the *R. helvetica* plasmid was included as positive control.

### Statistical comparison of sequencing methods

Chi-square test was performed to test the null hypothesis of no sensitivity differences between custom Sanger sequencing and real-time pyrosequencing. Sensitivity was calculated for all tick samples as well as tick samples divided into *gltA* copy numbers in the initial template. H0 was rejected if P ≤ 0.05.

## Competing interests

The authors declare that they have no competing interests.

## Authors’ contributions

CS conceived the study and designed the oligonucleotides. EJ and SS carried out the laboratory experiments. EJ and CS drafted the manuscript. All authors participated in data analysis, and read and approved the final manuscript.

## Pre-publication history

The pre-publication history for this paper can be accessed here:

http://www.biomedcentral.com/1471-2334/12/261/prepub
